# Comparative *In Vitro* Evaluation of Human Dental Pulp and Follicle Stem Cell Commitment

**DOI:** 10.22074/cellj.2016.4727

**Published:** 2016-09-26

**Authors:** Razieh Karamzadeh, Mohamadreza Baghaban Eslaminejad, Ali Sharifi-Zarchi

**Affiliations:** Department of Stem Cells and Developmental Biology, Cell Science Research Center, Royan Institute for Stem Cell Biology and Technology, ACECR, Tehran, Iran

**Keywords:** Dental Pulp, Stem Cells, Development, Genes

## Abstract

**Objective:**

Pulp and periodontal tissues are well-known sources of mesenchymal stem cells (MSCs) that provide a promising place in tissue engineering and regenerative medicine. The molecular mechanisms underlying commitment and differentiation of dental stem cells that originate from different dental tissues are not fully understood. In this study, we have compared the expression levels of pluripotency factors along with immunological and developmentally-related markers in the culture of human dental pulp stem cells (hDPSCs), human dental follicle stem cells (hDFSCs), and human embryonic stem cells (hESCs).

**Materials and Methods:**

In this experimental study, isolated human dental stem cells were investigated using quantitative polymerase chain reaction (qPCR), immunostaining, and fluorescence-activated cell sorting (FACS). Additionally, we conducted gene ontology (GO) analysis of differentially expressed genes and compared them between dental stem cells and pluripotent stem cells.

**Results:**

The results demonstrated that pluripotency (*OCT4* and *SOX2*) and immunological (*IL-6* and *TLR4*) factors had higher expressions in hDFSCs, with the exception of the *JAGGED-1/NOTCH1* ratio, *c-MYC* and *NESTIN* which expressed more in hDPSCs. Immunostaining of OCT4, *SOX2* and c-MYC showed cytoplasmic and nucleus localization in both groups at similar passages. GO analysis showed that the majority of hDFSCs and hDPSCs populations were in the synthesis (S) and mitosis (M) phases of the cell cycle, respectively.

**Conclusion:**

This study showed different status of heterogeneous hDPSCs and hDFSCs in terms of stemness, differentiation fate, and cell cycle phases. Therefore, the different behaviors of dental stem cells should be considered based on clinical treatment variations.

## Introduction

Dental stem cells are isolated from primary as well as permanent teeth during the developmental stages of tooth organogenesis. These cells are considered to be a tremendous source for repair and regeneration of both dental and/or non-dental tissues because of their accessibility, high proliferative ability, and multi-lineage differentiation capacity; additionally, they possess mesenchymal stem cell (MSC) features such as immunoregulatory activities (1,2). Dental stem cell fate commitment and self-renewal are under the control of diverse mechanisms that involve regulation of both transcription factors and signaling pathway components (3,4). 

Pluripotent transcription factors OCT4 and SOX2 along with c-MYC and KLF4 play important roles not only in embryonic stem cells (ESC) self-renewal and differentiation capacity, but also in somatic cell reprogramming which result in the generation of induced pluripotent stem cells (iPSCs). These factors are involved in regulation of the pluripotent stem cell cycle which is considered to be one of the key factors in the maintenance of pluripotency (5). Some somatic stem cell populations including germ cells, neural stem cells and MSCs also express low amounts of pluripotency factors. In human dental-related tissues, both human dental pulp stem cells (hDPSCs) and human dental follicle stem cells (hDFSCs) express these pluripotency markers which include OCT4, NANOG, and SOX2 (6,7). Proteomic analysis of hDPSCs during differentiation shows that self-renewal and differentiation regulatory genes are associated with other signaling pathways such as Notch, transforming growth factor beta (TGFβ), bone morphogenic protein (BMP) and Wnt (8,9). 

Notch signaling is involved in a variety of cellular processes such as stem cell survival and proliferation as well as cell-fate determination and apoptosis. Inhibition or activation of this pathway during stem cell division results in the initiation of cell commitment into a specific progenitor (10). Notch signaling also affects tissue homeostasis by regulating the expression of inflammatory molecules such as interleukin-6 (IL-6) (11,13). NOTCH1 and NESTIN have been primarily identified as markers of dental follicle cells (14). However, these markers are also expressed in hDPSCs and it has been demonstrated that upregulation of NESTIN is involved in dental pulp cell differentiation into functional odontoblasts (15). 

In this study, we investigated a combination of central factors considered to be involved in dental follicle and pulp stem cell fate commitment and differentiation using experimental and bioinformatic analysis. Although studies have researched these factors individually, our intention was to perform a combined comparative evaluation of the factors in two dental-derived stem cells versus pluripotent stem cells, which have not been previously investigated. Despite the close functions of these factors during development, we divided these factors into three categories in terms of involvement in different stem cell status: i. Pluripotency factors (OCT4, SOX2, and cMYC), ii. Dental stem cell developmentally-related components (NOTCH1, JAGGED-1, and NESTIN), and immunological markers such as mesenchymal/ hematopoietic and perivascular markers plus IL-6 and toll-like receptor 4 (TLR4). 

## Materials and Methods

### Cell isolation, culture and characterization

This was an experimental study. The study protocol received the approval of the Institutional Review Board (IRB) of Royan Institute, Tehran, Iran. Human impacted third molars (n=6) were extracted from healthy young adults (20-30 years) who underwent orthodontic treatment after obtaining informed consent from each patient. hDPSCs (n=3) and hDFSCs (n=3) were obtained from dental pulp and dental follicle by enzymatic digestion as previously described (14,16,17). Briefly, dental pulp and follicle tissues were digested with Dispase (Life Technologies, USA) and Collagenase I (Sigma, USA) and cultured in minimum essential medium alpha (α-MEM, Life Technologies, USA) supplemented with 10% (v/v) heat inactivated fetal bovine serum B (Life Technologies, USA), 100 U/mL penicillin G, 100 mg/mL streptomycin (Life Technologies, USA), and 100 µM L-ascorbic acid 2-phosphate (Sigma, USA), 2 mM L-glutamine (Life Technologies, USA), and 0.25 mg/ml amphotericin B (Life Technologies, USA) in a 37˚C incubator with 5% CO_2_ . The culture medium was changed every 3 days and cells were harvested by trypsin digestion for further analysis. The cell morphology images were captured under an inverted microscope (Olympus, Japan). All experiments were performed at passage 3 (P3) except karyotyping which was performed at passage 4 (P4) for longer chromosomal evaluation of numerical and structural aberrations. 

For karyotype analysis, the cells were treated with thymidine (Sigma, USA) for 12 hours at 37˚C, washed with fresh medium, and treated with colcemid (Life Technologies, USA) for an additional 30 minutes. Then, trypsinized cells were swollen with KCl and fixed in ice-cold methanol and acetic acid (3:1). After dropping the cells onto chilled slides, the chromosomes were visualized using standard G-band staining. Additional analysis was performed using CytoVision software. We evaluated at least 30 metaphase spreads and five banded karyotypes for chromosomal analysis according to the International System for Human Cytogenetic Nomenclature (ISCN). 

In order to confirm multipotency, hDPSCs and hDFSCs were differentiated towards osteoblast, adipocyte, and chondrocyte cell lineages as previously described (18) and differentiation capacities confirmed by alizarin red S, oil red O, and toluidine blue (Sigma, USA) staining for bone, cartilage, and fat differentiation, respectively. Reverse transcription-polymerase chain reaction (RT-PCR) was also used for further validation. 

Briefly, for osteogenic differentiation, cells were cultured at 5000 cells/well in 6-well plates. At 50% confluency, we replaced the medium with osteogenic medium that consisted of α-MEM supplemented with 0.2 mM L-ascorbic acid 2-phosphate (Sigma, USA), 0.01 µM dexamethasone (Sigma, USA), and 10 mM β-glycerol phosphate (Sigma, USA). In order to verify adipogenic differentiation, the cells were cultured at 5000 cells/well in 6-well plates and allowed to grow to 50% confluency. The medium was then replaced with adipogenic medium that consisted of α-MEM supplemented with 1 mM dexamethasone and 60 mM indomethacin (Sigma, USA). For chondrogenic differentiation, P3 cells were placed in a conical tube, pelleted by 400 g centrifugation, provided with chondrogenic medium, and incubated in an atmosphere of 5% CO_2_ and 37˚C. The chondrogenic medium consisted of hMSC Chondrogenic SingleQuots (Lonza Walkersville, Inc., USA) supplemented with 10 ng/ml TGF-β3 (Peprotech, UK). The cultures were maintained for 3 weeks with twice weekly medium changes. At the end of the maintenance period, the pellets were fixed in 4% paraformaldehyde for a day and prepared for microtomy using standard protocols. 

### Fluorescence-activated cell sorting analysis

Fluorescence-activated cell sorting (FACS) analysis was performed for hDPSCs and hDFSCs (P3) according to the manufacturer’s protocols using FITC or phycoerythrin (PE)-conjugated antibodies CD31, CD105, CD73, CD11b, CD44, and CD146 (all from BD-Pharmingen, USA); CD45, CD34 (eBioscience, USA); and CD90 (Dako, Denmark). A FACSCalibur flow cytometer (BD Bioscience, USA) was used for the analyses. Positive cells were identified by comparison with the corresponding isotype controls. 

### Quantitative polymerase chain reaction analysis

Total RNA was extracted from undifferentiated (P3) and differentiated hDPSCs and hDFSCs using TRIzol (Sigma, USA). DNase treatment (Thermo Scientific, Germany) and first strand cDNA synthesis was performed according to the manufacturer’s protocol using a TaKaRa PrimeScript™ RT Reagent Kit (TaKaRa, Japan). PCR amplification was carried out with Platinum® Blue PCR SuperMix (Life Technologies, USA). 

Quantitative polymerase chain reaction (qPCR) was performed according to the manufacturer’s protocol (Applied Biosystems, USA). The expressions of all genes were compared with ESCs as the external control. The amount of transcripts was normalized with *GAPDH*. We used the delta delta cycle threshold (ΔΔCT) method to calculate the relative fold change in gene expression. 

### Immunofluorescence

Both hDPSCs and hDFSCs were cultured in chamber slides and the immunofluorescence procedure for SOX2, c-MYC, OCT4, and NESTIN (all from Santa Cruz Biotechnology, USA) with secondary antibodies, Alexa Fluor^®^ 488 (Life Technologies, USA) and FITC (Sigma, USA) were performed according to the manufacturer’s protocol. 

### Bioinformatics analysis

High-throughput transcriptome profiles of the hDFSCs, hDPSCs, hiPSCs, and human ESCs (hESCs) were obtained from the Gene Expression Omnibus (GEO). The similarity of microarray platforms used to generate all data improved the reliability of crosscomparison of the data from different cell types. The datasets were log2 scaled and quantile normalized. Differentially expressed genes were identified under the criteria of a minimum two log-fold change and P<0.01. The gene ontology (GO) analysis of the differentially expressed genes was performed using David (19). The GO terms with adjusted (Benjamini and Hotchberg) P<0.01 were assumed as significant in our analysis. 

### Statistical analysis

Data from the experiments were expressed as mean ± SEM of at least three independent experiments. Statistical significance was determined by the paired Student’s t test and one-way ANOVA using R programming, in which we considered P<0.05 to be significant. 

## Results

### Characterization of human dental pulp stem cells and human dental follicle stem cells

**Fig.1:**
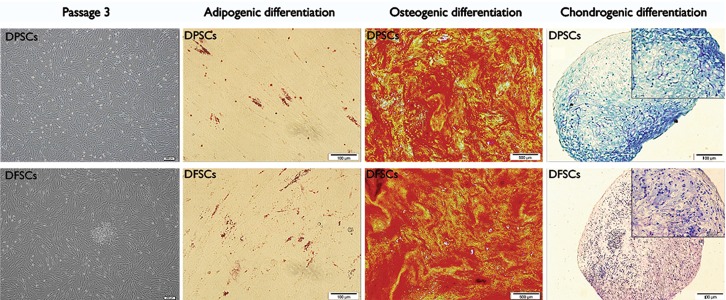
Human dental follicle stem cell (hDFPC) and human dental pulp stem cell (hDPSC) morphology and differentiation potential in osteoblasts, adipocytes, and chondrocytes. These results indicated that both groups possess mesenchymal stem cell (MSCs) properties in terms of differentiation capacity.

**Fig.2:**
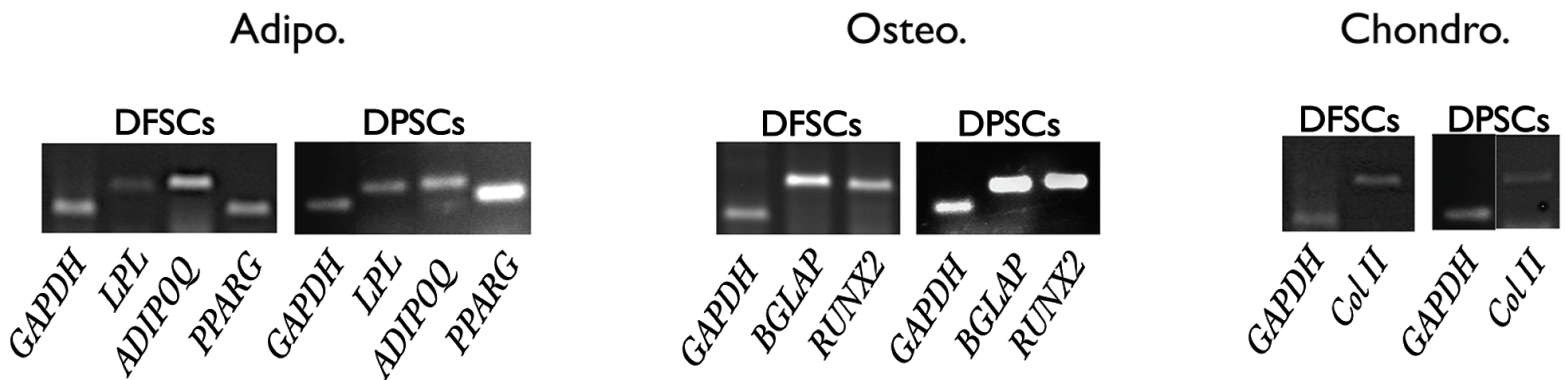
Human dental follicle stem cell (hDFPC) and human dental pulp stem cell (hDPSC) differentiation markers towards osteoblast, adipocyte, and chondrocyte differentiation. Reverse transcription-polymerase chain reaction (RT-PCR) results indicated the expressions of differentiation genes compared to the housekeeping gene (GAPDH).

hDPSCs and hDFSCs appeared as plastic adherent cells that had a fibroblast-like morphology (Fig.1). The cells successfully differentiated into bone, adipose, and cartilage cells as evidenced by alizarin red S, oil red O, and toluidine blue staining (Fig.1). RT-PCR results confirmed expressions of osteogenic (*RUNX2* and *PGLAP*), adipogenic (*LPL, PPARG* and *ADIPOQ*), and chondrogenic (*COL2A1*) genes (Fig.2). 

**Fig.3:**
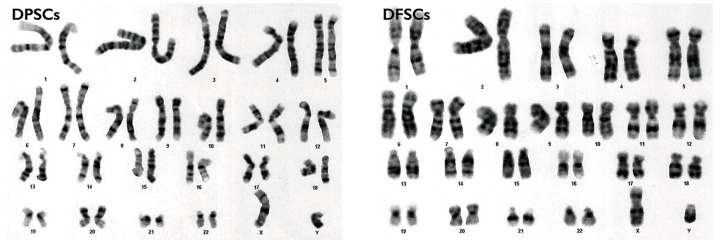
Karyotyping results of human dental pulp stem cell (hDPSCs) and human dental follicle stem cells (hDFSCs). The results did not reveal any numerical changes in chromosome numbers according to the International System for Human Cytogenetic Nomenclature (ISCN).

Karyotype analyses of both groups by size and position did not reveal any numerical changes in chromosome numbers on the basis of GTG tech at 350 band resolution (Fig.3). 

### Comparative expressions of mesenchymal, hematopoietic, and pre-vascular marker CD146 

**Fig.4:**
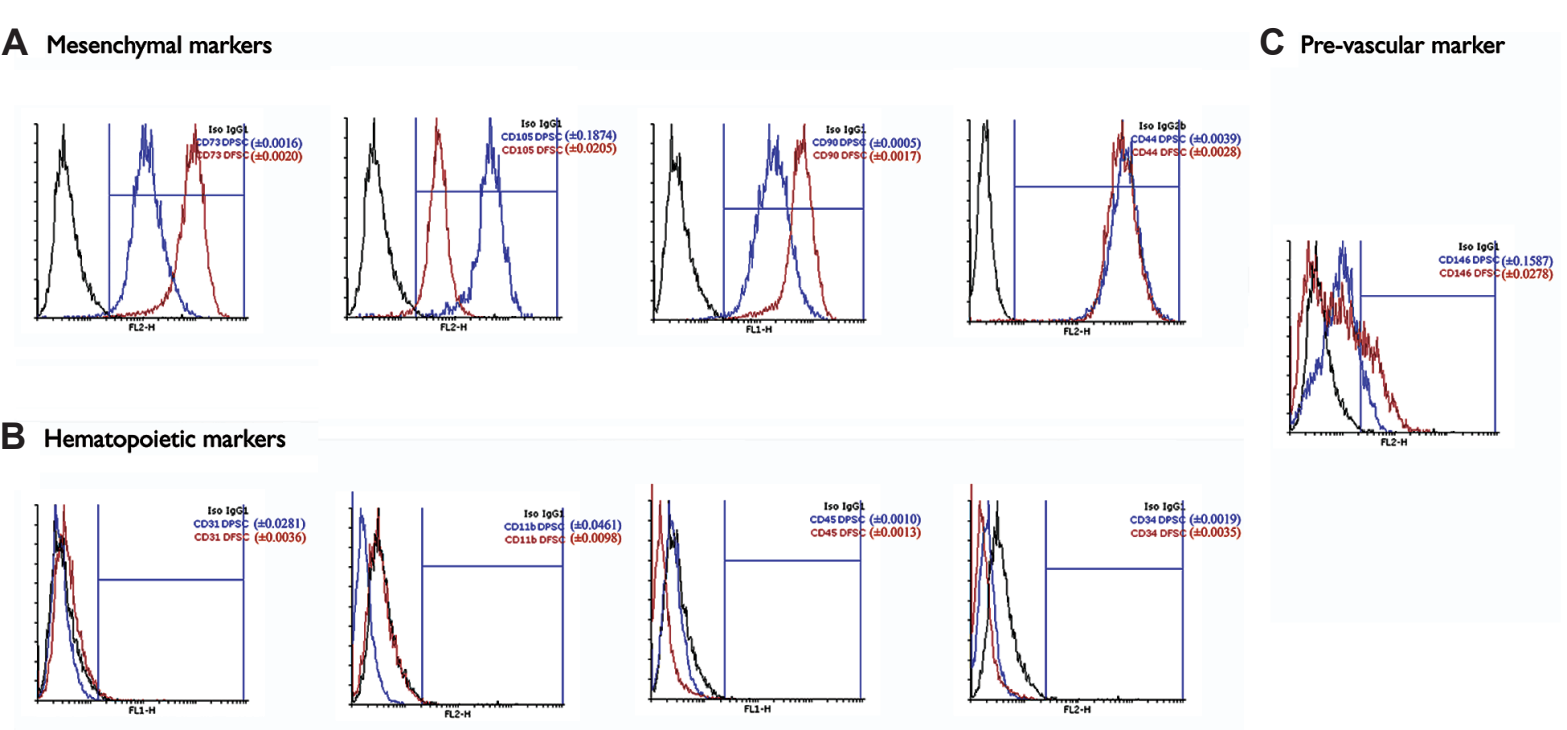
Immunophenotyping results of the expressions of A. Mesenchymal, B. Hematopoietic, and C. Premarkers in human dental follicle stem cells (hDFSCs) and human dental pulp stem cells (hDPSCs). The results have shown that both groups possess mesenchymal stem cell (MSC) properties in terms of conventional surface markers.

FACS analysis for the expressions of mesenchymal stromal markers showed positive expressions of CD90, CD73, CD44, and CD105 (approximately 90% positive cells) and negative expressions of hematopoietic markers CD34, CD45, and CD11b in both groups. CD146 showed the only significant variation with higher expression in hDFSCs compared to hDPSCs (Fig.4). 

### Comparative mRNA expression of pluripotecy factors along with developmental and immunological markers

**Fig.5:**
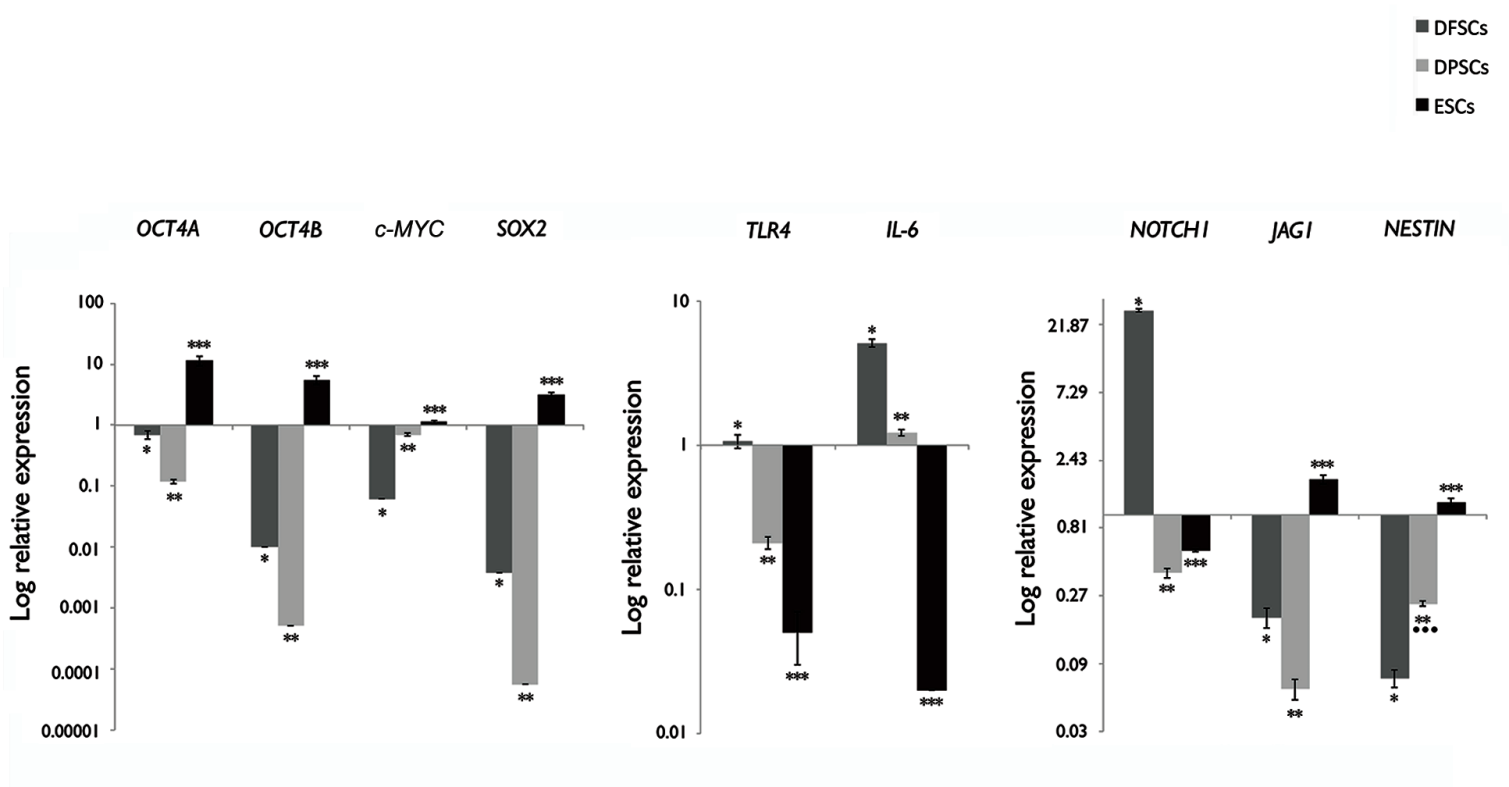
Quantitative real-time polymerase chain reaction (qRT-PCR) results of pluripotency (*OCT4A/B, c-MYC* and *SOX2*), immunological (*IL-6* and *TLR4*), and developmental factors [*NOTCH1, JAG1,* and *NESTIN*] in human dental pulp stem cells (hDPSCs), human dental follicle stem cells (hDFSCs) and human embryonic stem cells (hESCs) with *GAPDH* as the internal control (n=3). These results showed that pluripotent factors had higher expression in hDFSCs (except for *c-MYC*). Immunological markers had higher expression in hDFSCs. In the case of developmentally-related genes *NOTCH1* and *JAG1* had higher expression in hDFSCs compared to hDPSCs.

Comparative mRNA expression of pluripotency factors *OCT4, SOX2* and *c-MYC* as well as developmental markers *NOTCH1* and *JAGGED-1* indicated significantly higher expressions of OCT4, and SOX2 in hDFSCs compared with hDPSCs (P<0.05). In this regard there was a higher *JAG1/ NOTCH1* ratio in hDPSCs compared to hDFSCs (Fig.5). 

Evaluation of OCT4 isoforms indicated that expressions of *OCT4A* and *OCT4B* in both groups were higher in hDFSCs compared to hDPSCs. *OCT4A* had higher level of expression compared to *OCT4B* in both groups (P<0.05). On the other hand, there was a higher expression of *c-MYC* observed in hDPSCs compared to the hDFSCs (Fig.5). For confirmation, hESCs were considered as the external control. 

qPCR analysis indicated a significantly lower expression of the early neural stem cell marker *NESTIN* in hDFSCs compared to hDPSCs (P<0.05). In contrast, we observed significantly lower expressions of *IL-6* and *TLR4* in hDPSCs compared to hDFSCs (P<0.05, Fig .5). 

### Protein expression and subcellular localization of OCT4, SOX2, c-MYC and NESTIN

**Fig.6:**
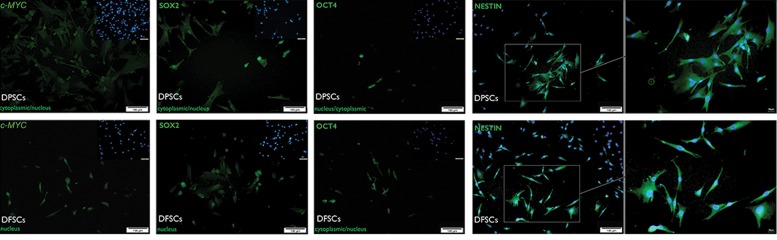
Immunocytofluorescence results of OCT4, c-MYC, SOX2 and NESTIN expressions in human dental pulp stem cells (hDPSCs) and human dental follicle stem cells (hDFSCs). Cell nuclei were stained with DAPI as indicated in the upper-right side of each section (c-MYC, SOX2, and OCT4) and also merged in the case of cytoplasmic NESTIN expression (magnification bar: 100 µm).

Immunostaining showed the expressions of OCT4, SOX2 and c-MYC in hDFSCs and hDPSCs. In both groups, although proteins were present in the cytoplasm and nucleus of cells, we observed more proteins in the cytoplasm of hDPSCs (data not shown). Although there was NESTIN expression at the protein level in both groups, it did not significantly differ (P>0.05, Fig .6). 

### Gene ontology of differentially expressed genes 

**Fig.7:**
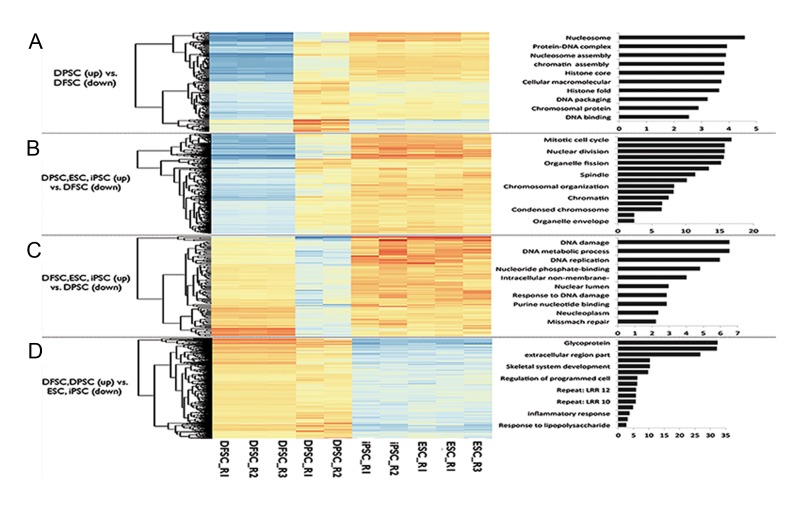
Heat map of differentially expressed genes which A. Upregulated in human dental pulp stem cells (hDPSCs) and downregulated in human dental follicle stem cells (hDFSCs), B. Upregulated in hDPSCs, human embryonic stem cells (hESCs), and human induced pluripotent stem cells (hiPSCs) versus downregulated in hDFSCs, C. Upregulated in hDFSCs, hESCs, and hiPSCs versus downregulated in hDPSCs, and D. Upregulated in hDFSCs and hDPSCs versus downregulated in hESCs and hiPSCs. (R: replicate).

Comparative functional clustering of differentially expressed hDFSC and hDPSC genes that most differentially upregulated genes in hDPSCs compared to hDFSCs were related to nucleosome and nucleosome assembly (Fig.7A). Clustering of differentially expressed genes of each group (hDFSCs or hDPSCs) with pluripotent stem cells (hESCs and hiPSCs) also confirmed these findings (Fig.7B,C). As shown in Figure 7B, most differentially upregulated genes in DPSCs and pluripotent stem cells compared to the hDPSCs group were related to the mitosis (M) phase of the cell cycle (i.e., mitotic cell cycle, nuclear division, and chromosomal organization, Fig .7B). However differentially upregulated genes in hDFSCs and pluripotent stem cells compared to the hDFSCs group were associated with the S phase of the cell cycle (i.e., DNA replication and DNA metabolic processes, Fig .7C) GO results of differentially upregulated genes in dental versus pluripotent stem cells (Fig.7D) indicated that the majority of these genes were related to the extracellular region and immunological-related factors involved in inflammatory and immune responses. 

## Discussion

In this study, we comparatively evaluated three groups of central elements-pluripotency factors, developmentally-related components, and immunological markers in two sources of pulp and follicle MSCs, which have not been investigated by this aim. Our findings demonstrated significant expressions of these factors at the same passages which might impact the distinct developmental status of these cells. 

Recent studies demonstrated the existence of different epigenetic mechanisms in differentiation of dental pulp and follicle stem cells. The relationship between expression of pluripotent factors and cell passages was also reported (4). In this regard, hDPSCs displayed a higher expression of pluripotency marker OCT4 compared to hDFSCs (7). In contrast, as indicated in the Results section, our findings showed lower expressions of *SOX2* and *OCT4* in a heterogeneous population of hDPSCs compared to hDFSCs. The discrepancy in findings might be due to the different passages in which the analysis was performed. While we compared the cells at the same passage (P3), others performed their studies with cells from different passages. 

We also investigated the expression of *OCT4* isoforms in order to differentiate isoform expression in both cells compared to hESCs. To the best of our knowledge there no experiment has compared different *OCT4* isoforms in hDPSCs and hDFSCs. Interestingly, we found higher expression of *OCT4A* in both cells compared with *OCT4B*. According to evidence, different localization patterns of the OCT4 isoforms might have been attributed to the subcellular specific isoform behaviors. In this regard, OCT4A acts as a nuclear protein while OCT4B is a cytoplasmic protein (20). Other studies have reported the possible cytoplasmic translocation of OCT4A, c-MYC and SOX2 by regulatory mechanisms during cellular passaging (4). Here, we demonstrated both the cytoplasmic and nucleus localization of OCT4, SOX2 and c-MYC in hDPSCs and hDFSCs. Although the localization pattern of OCT4, SOX2 and cMYC in hDPSCs has previously been reported, there was no data regarding hDFSCs. Research has shown the possibility of a different role for OCT4 in somatic stem cells compared with ESCs (21). It would be of interest to determine whether this localization pattern was involved in stemness status of these cells. In addition, according to our findings, we have observed comparatively higher expressions of *OCTA* and *SOX2* in hDFSCs than hDPSCs. Future research can investigate the possible correlations between the efficiency of iPSCs generation in these stem cells. 

It is well established that pluripotent factors are involved in cell cycle regulation (22). Studies indicate that hESCs spend 65% of their time in the synthesis (S) phase of the cell cycle, whereas induction of differentiation decreases the S phase and increases the G1 phase (23). This specification is one of the indicators of pluripotent stem cells which permit them to proliferate faster than differentiating stem cells. Our GO analysis of differentially expressed genes between hDFSCs, hDPSCs, and pluripotent stem cells indicate the distinct similarity along with different functions of pluripotent factors in somatic stem cell cycle regulation. Further investigations are essential to explore the exact relation of pluripotent factors and cell cycle regulation in somatic stem cells in order to discriminate the role of pluripotent factors in these cells. 

Notch signaling plays an important role in cell fate determination by influencing proliferation, differentiation and apoptosis events (24). The ligand/receptor ratio in the notch signaling pathway in multicellular systems indicates sending or receiving signaling potential of a cell population to stay or differentiate into progenitor cells (25). Accordingly, our results have shown a higher JAGGED-1/NOTCH1 ratio in hESCs. This indicated the tendency of these cells to remain in a default undifferentiated fate status known as the sending potential which is in agreement with previous findings about the induction of ES cell differentiation into mesodermal cell lineages due to the higher amount of notch receptors (26). In both hDPSCs and hDFSCs, we observed lower JAGGED-1/NOTCH1 ratios compared to the ESCs, which was known as receiving potential. This might explain the fate restriction potential of somatic versus ESCs. According to our findings a higher *JAGGED-1/NOTCH-1* ratio existed in hDPSCs compared with hDFSCs. This implied that although the heterogeneous hDFSC population expressed higher amounts of pluripotent transcription factors, they were more ready for cell fate switching compared to hDPSCs (standby fate status). 

We found higher expressions of pro-inflammatory cytokine *IL-6* in hDFSCs compared to hDPSCs. This might be due to the regulatory function of notch signaling (NOTCH1 receptor) on the activation of some inflammatory cytokines in hDFSCs. In addition, it has been demonstrated that IL-6 expression was under control of the TLR4 signaling pathway; higher expression of TLR4 along with IL-6 indicated the functional role of TLR4 as an innate immune receptor in hDFSCs and its pivotal correlation in stem cell commitment and immunity (27). 

CD146 is a cell adhesion molecule strictly regulated during development (28). It has been demonstrated that presence of this putative MSC marker provides a higher tri-lineage differentiation potential in MSCs (29). The higher expression of CD146 in hDFSCs than hDPSCs along with different expressions of notch signaling component and pluripotency factors may also confirm the standby fate status in hDFSCs. According to evidence, upregulation of NESTIN during hDPSCs odontogenic differentiation (15) and higher expression of NESTIN transcripts in hDPSCs compared to hDFSCs shows the odontogenic differentiation potential of these cells, which agrees with other observations. 

## Conclusion

In conjunction, we investigated number of critical factors involved in dental stem cell fate restriction and differentiation. These results showed that heterogeneous hDPSCs might be more developmentally restricted compared to hDFSCs by expression of lower amounts of pluripotent/reprogramming factors (*OCT4, SOX2,* and *CD146*) and higher levels of a developmental factor ratio, *JAGGED-1/NOTCH1*. Discrimination of such factors might improve future clinical applications of somatic stem cells for vital pulp therapy and tissue engineering. 
